# Artificial Intelligence and ECG: A New Frontier in Cardiac Diagnostics and Prevention

**DOI:** 10.3390/biomedicines13071685

**Published:** 2025-07-09

**Authors:** Dorota Bartusik-Aebisher, Kacper Rogóż, David Aebisher

**Affiliations:** 1 Department of Biochemistry and General Chemistry, Medical College, The Rzeszów University, 35-310 Rzeszów, Poland; dbartusikaebisher@ur.edu.pl; 2 English Division Science Club, Medical College, The Rzeszów University, 35-310 Rzeszów, Poland; kr117626@stud.ur.edu.pl; 3 Department of Photomedicine and Physical Chemistry, Medical College, The Rzeszów University, 35-310 Rzeszów, Poland

**Keywords:** artificial intelligence, electrocardiography, wearable devices, smartwatch, atrial fibrillation, digital cardiology

## Abstract

**Objectives**: With the growing importance of mobile technology and artificial intelligence (AI) in healthcare, the development of automated cardiac diagnostic systems has gained strategic significance. This review aims to summarize the current state of knowledge on the use of AI in the analysis of electrocardiographic (ECG) signals obtained from wearable devices, particularly smartwatches, and to outline perspectives for future clinical applications. **Methods**: A narrative literature review was conducted using PubMed, Web of Science, and Scopus databases. The search focused on combinations of keywords related to AI, ECG, and wearable technologies. After screening and applying inclusion criteria, 152 publications were selected for final analysis. **Conclusions**: Modern AI algorithms—especially deep neural networks—show promise in detecting arrhythmias, heart failure, prolonged QT syndrome, and other cardiovascular conditions. Smartwatches without ECG sensors, using photoplethysmography (PPG) and machine learning, show potential as supportive tools for preliminary atrial fibrillation (AF) screening at the population level, although further validation in diverse real-world settings is needed. This article explores innovation trends such as genetic data integration, digital twins, federated learning, and local signal processing. Regulatory, technical, and ethical challenges are also discussed, along with the issue of limited clinical evidence. Artificial intelligence enables a significant enhancement of personalized, mobile, and preventive cardiology. Its integration into smartwatch ECG analysis opens a path toward early detection of cardiac disorders and the implementation of population-scale screening approaches.

## 1. Introduction

With the rapid development of mobile technology and artificial intelligence (AI), healthcare is entering a new era—one defined by remote and automated medical services. Cardiovascular diseases, which, according to the World Health Organization, account for the largest number of deaths worldwide, are becoming a priority area for the deployment of intelligent diagnostic systems, such as remote heart rhythm monitoring and ECG analysis using AI [1]. Early detection of cardiac disorders significantly improves the prognosis and quality of life of patients [2].

This article presents the application of mobile automated cardiac care systems, with a focus on AI-assisted smartwatches. Non-invasive acquisition of physiological signals—including photoplethysmogram (PPG), electrocardiogram (ECG), phonocardiogram (PCG)—and their analysis based on artificial intelligence and deep learning enable predictive diagnosis of cardiovascular anomalies and diseases [3]. At the same time, the successful implementation of such systems requires not only accurate algorithms but also their interpretability (Explainable AI), integration into clinical practice, and evaluation of usability at population and systemic scales [4]. This article analyzes the current state of the art and identifies implementation challenges and possible directions for further research and innovation in this rapidly developing area.

## 2. Materials and Methods

This article presents a narrative review of the current literature on the use of artificial intelligence in ECG analysis from wearable devices. The initial stage of the literature search was conducted using the PubMed, Web of Science, and Scopus databases, using the following keyword combinations: “smartwatch AND ECG AND artificial intelligence”, “smartwatch AND ECG AND AI”. This step resulted in a total of 42 and 22 results (PubMed), 31 and 18 results (Web of Science), and 24 and 17 results (Scopus). A total of 154 publications were obtained, of which 69 unique items were left for further analysis after removal of duplicates.

In the second stage, an extended search was carried out, taking into account broader and related subject headings, such as: “ECG AND artificial intelligence”, “smartwatch AND ECG”, “wearable AND ECG AND AI”, “digital health AND arrhythmia detection”, “artificial intelligence AND atrial fibrillation AND wearable”, “AI AND ECG-based diagnosis”, “deep learning AND ECG”. The aim of the second phase was to expand the scope of the review to capture studies not included in the first phase, but relevant to the application of AI in the analysis of ECG signals from smartwatch devices.

**Figure 1:**
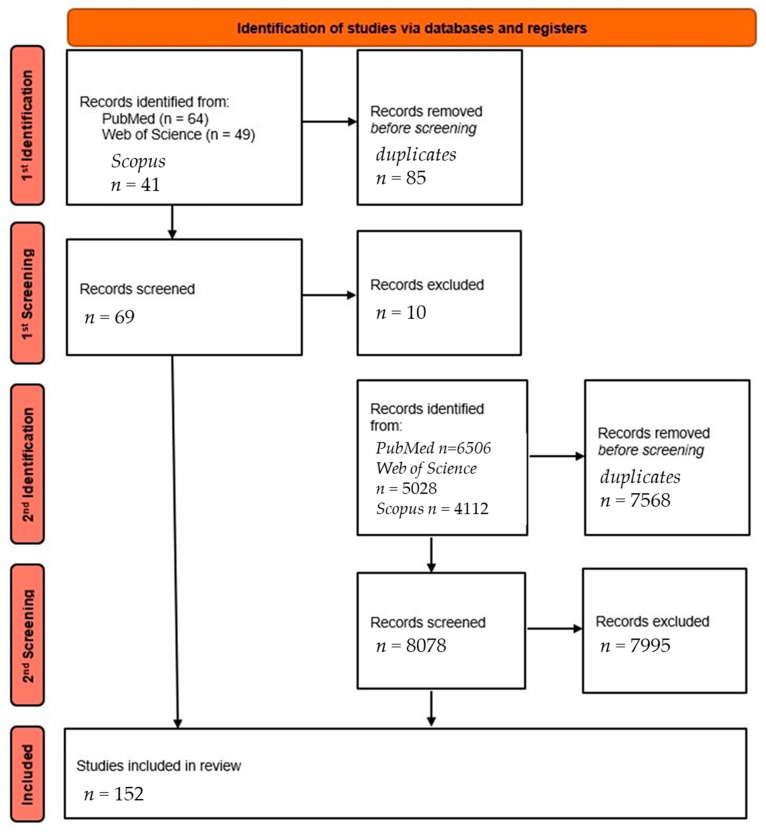
Literature selection process (PRISMA-style adapted flowchart for narrative review) [own elaboration].

After applying similar inclusion criteria—primarily original clinical studies (both prospective and retrospective), meta-analyses, publications in English from the last ~5 years—and excluding papers not directly related to the topic, 152 publications were finally selected for analysis. Figure 1 presents the literature selection process using an adapted PRISMA-style flowchart, tailored to the narrative review methodology.

## 3. AI in ECG Analysis

Artificial intelligence has great potential in cardiology, especially in ECG analysis. AI supports the interpretation of ECG results, especially deep learning (DL) methods such as convolutional neural networks (CNNs), which can effectively diagnose heart disease. These models support clinicians by reducing the time to diagnosis of arrhythmias and facilitating the daily monitoring of patients, especially when using mobile devices [5]. AI also has applications in ECG signal quality assessment, artefact detection, and automatic rejection of disturbed leads, which increases the reliability of analysis, especially in wearable devices and telemedicine [6,7]. Table 1 summarizes the use of selected AI algorithms in ECG analysis.

It is common to combine two or more AI architectures to achieve synergistic effects, such as integrating long short-term memory (LSTM) networks with convolutional neural networks (CNNs) [21].

AI can predict AF risk in a similar way to classical clinical factors, even without knowledge of patient data—just based on the classical 12-lead ECG. However, combining AI with clinical data produces the most accurate results. Nonetheless, AI can serve as a rapid, automated screening tool [23]. Even with an apparently normal ECG with sinus rhythm, AI models are able to detect invisible traces of AF, such as P-wave changes or microfluctuations in rhythm. This opens up a potential avenue for earlier detection of disease-related markers, although prospective validation is required before routine clinical implementation [24]. 

Artificial intelligence is increasingly entering cardiac electrophysiology, going far beyond the analysis of the surface ECG. As the review by Cersosimo et al. shows, AI is finding applications in the analysis of intracardiac electrograms, in the risk assessment of sudden cardiac arrest (SCD), and in the planning of ablation procedures. The authors also showed how large language models, such as ChatGPT-4, can support clinicians in interpreting complex electrophysiological data and translating them into clinical decisions [25]. In contrast, an expert consensus from the European Heart Rhythm Association (EHRA), the Heart Rhythm Society (HRS), and the European Society of Cardiology (ESC), published by Svennberg et al., identifies three main areas of application for AI in electrophysiology: detection of rhythm disturbances (including subclinical atrial fibrillation), support for ablation planning, and prediction of treatment outcomes. Importantly, more than half of the studies included in the review showed improvements in diagnostic accuracy or treatment efficacy through AI integration [26]. Multimodal approaches, combining ECG data with cardiac imaging (e.g., MRI or CT), are also increasingly important. Work by Tang et al. has shown that such hybrid models allow better localization of arrhythmia sources, which increases the effectiveness of ablation, especially for complex rhythm disorders such as atrial fibrillation or ventricular tachycardia [27]. The accumulated evidence confirms that AI is no longer just an adjunctive tool but is becoming an integral part of modern cardiac electrophysiology—supporting diagnosis, treatment planning AND monitoring treatment outcomes, and ultimately improving patient prognosis.

Several recent studies have demonstrated that AI-ECG can translate into tangible clinical benefits. In a multicenter randomized trial, Lin et al. showed that AI-generated ECG alerts led to earlier clinical intervention, resulting in a 17% reduction in all-cause mortality and a 93% reduction in cardiac mortality [28]. Furthermore, the cluster-randomized EAGLE trial (Yao et al.) in primary care (*n* = 22,641) showed that access to AI-ECG increased the early diagnosis of low ejection fraction (≤50%) from 1.6% to 2.1% (OR 1.32, *p* = 0.007), facilitating timely heart failure treatment [29,30]. These data support that AI-guided ECG not only refines diagnostics but also contributes to improved morbidity and mortality outcomes. Appropriate software (such as AccurBeat) further improves the accuracy and consistency of the data compared to manufacturers’ algorithms [31]. A systematic review by Popat et al. showed that AI models, including Artificial Intelligence-Enhanced Electrocardiogram (AI-ECG) and machine learning, have better sensitivity and specificity than traditional risk scales like CHADS-VASc, ARIC, or CHARGER-AF. However, there is still a lack of standardization of procedure and data consistency between studies [32]. 

For example, AI-ECG algorithms allow the early detection of prolonged QT syndrome (QTc)—crucial in the prevention of sudden cardiac death—and also enable the analysis of P-, R-, and T-wave alternans, i.e., alternations in the amplitude or shape of specific waves in the ECG recording. The latter is made possible by a combination of CNNs and the Modified Moving Average (MMA) method. This allows faster detection of risks such as atrial fibrillation or sudden cardiac death and more effective risk stratification [33,34]. AF is associated with stroke risk. The use of AI and ML to analyze ECG and biomarker data can help to better predict stroke risk and personalize an individualized treatment plan [35]. In addition, by analyzing routine 12-lead ECGs, AI is able to predict whether a patient may have a reduced ejection fraction of the heart and requires prompt and effective diagnosis, and in patients diagnosed with heart failure with reduced ejection fraction, it is able to predict annual mortality [30,36,37]. In one of the first clinical trials, the Viz HCM system successfully assisted physicians in identifying new cases of hypertrophic cardiomyopathy (HCM), enabling rapid diagnosis and confirmation of the disease in almost 8% of eligible patients [38]. Artificial intelligence may very well help to rule out valvular heart disease based on the ECG but does not replace the physician in its definitive diagnosis, due to its low positive predictive value (PPV) [15]. AI can also support ED physicians in the rapid and accurate detection of heart attacks [39]. The use of AI to analyze ECGs may represent a promising development in the diagnostic process, though further high-quality evidence is required to confirm its clinical equivalence to traditional diagnostic approaches. Also of interest seems to be the possibility of joint analysis of ECG and other diagnostic tests, such as Electroencephalogram (EEG) [40]. Hitherto unrelated parameters, in the context of AI analysis, may prove to be a useful diagnostic tool. In recent years, developments in federated learning have made it possible to train AI models on distributed ECG data from multiple institutions without the need for data transfer [41,42,43]. This is a significant step forward in terms of data privacy and model generalizability.

## 4. The Use of Wearable Devices Combined with Artificial Intelligence (AI)

### 4.1. Detection of Atrial Fibrillation (AF)

Thanks to applications like FibriCheck, which only use the PPG signal for pulse analysis, popular smartwatches and fitness bands without a native ECG system gain the ability to detect atrial fibrillation (AF) in semi-controlled conditions with high efficiency [44]. This suggests that low-cost and widely available equipment could serve as a preliminary screening option for AF, pending further clinical validation.

Improvements in pulse signal quality have also been achieved through basic machine learning techniques for correcting motion artefacts without the need for extensive hardware [45]. Alternatively, simpler traditional PPG signal cleaning, e.g., using wavelet filtering (DWT/IDWT), allows noise removal but proves effective only under patient resting conditions [46].

Although the sensitivity of smartwatch models is high, the specificity is still too low. In a review by Baca et al., the best overall performance was shown by the decision tree model, which had the best balance between sensitivity, specificity, and accuracy [47].

The advanced PPG2ECGps model employs a convolutional neural network and allows mapping the dynamics of the real ECG from the recorded PPG and thus allows a more precise diagnosis of arrhythmias. This method still needs to be optimized and validated before it can reach wide clinical practice or mass use in smartwatches [48]. In addition, it has been shown that, in the case of atrial fibrillation, a PPG-based smartwatch can measure the average heart rate quite accurately every minute—especially with a heart rate <110 bbm and little patient activity [49].

The large THOFAWATCH study used a smartwatch to record a single ECG lead, without AI support. Huette et al. showed that even such a simple method can be useful in detecting postoperative atrial fibrillation (POAF) after thoracic surgery [50].

A single-lead ECG using an AI-assisted smartwatch can be a useful mobile tool, e.g., for recording episodes of atrial fibrillation (AF) in patients outside the doctor’s office, but the algorithm itself proves inferior in interpreting the results compared to the doctor. Such a result was shown in a study by Badertscher et al. based only on the Withings Scanwatch [51]. Another study by Mannhart et al., using a deep neural network (DNN) algorithm and five different smartwatches (Apple, Fitbit, AliveCor, Samsung, Withings), showed that artificial intelligence can substantially reduce the number of unreadable records and maintain high accuracy, making it a viable support in healthcare [52]. DNNs such as PulseAI significantly reduce ambiguous or indeterminate results, thus approaching the accuracy of physicians’ interpretations [53]. The Siamese network is an example of a personalized ML model using a single-channel ECG. Using pre-prepared ECG data sets with AF, training, and fine-tuning, based on a few short excerpts of a given patient, it could be prepared to detect AF and did so with almost maximum efficiency, dealing also with noise [54]. In the article by Jaeger et al., a comparison of three open-source algorithms for automatic ECG delineation (ECGdeli, ECGKit, NeuroKit) was performed on data collected from two classical databases. The analysis showed that the Apple Watch data had higher signal quality and greater sensitivity for feature point detection. The ECGdeli algorithm proved to be the most stable and versatile, while NeuroKit provided the highest temporal accuracy of measurements [55]. ML and especially deep learning algorithms can significantly improve the accuracy of diagnoses in detecting atrial arrhythmias using electrocardiograms from watches. DNNs are a better tool than the Apple app and can become a tool in the diagnosis of heart disease, helping clinicians to make faster and more precise clinical decisions [56,57,58]. In a comparison with the Holter ECG, the Amazfit smartwatch with its PPG algorithm and single-lead ECG has been shown to have very good sensitivity and agreement with the Holter in assessing atrial fibrillation, even those lasting >5 min and <60 min. The main problem is the moderate specificity, i.e., the risk of false alarms [59]. The CardioWatch 287-2 device, on the other hand, demonstrated sensitivity and specificity close to 100% and detected more cases of AF compared to a traditional Holter ECG due to longer monitoring [60]. This suggests potential clinical utility of wearable devices in this area, although broader studies across diverse populations are necessary to confirm these findings. A comparison of a smartwatch to an implantable AF monitoring device showed that Apple Watch + KardiaBand + AI appears to be a promising tool for long-term cardiac rhythm monitoring [61].

### 4.2. Assessment of Ejection Fraction (EF) and Heart Failure (HF)

Furthermore, AI based on the home ECG from a watch can detect heart failure with low ejection fraction (EF ≤ 40%) with relatively good accuracy [62]. Based on a single lead, AI models are able to estimate the risk of heart failure [63]. Daily ECGs from a smartwatch analyzed by AI can likely detect deterioration of heart failure at a very early stage, allowing doctors to respond more quickly and reduce the risk of hospital readmissions. In their current study, Lee et al. controlled the condition of patients after discharge using AI to analyze simple smartwatch data [64].

### 4.3. Assessment of QTc Distance and Electrolytic Disturbance

With the help of machine learning (ML), based on the analysis of the ECG signal, it is possible to accurately monitor and also distinguish between long QT syndrome genotypes [65,66,67]. Among patients with end-stage renal disease (ESRD), regular monitoring of blood potassium cation levels is very important. It appears that AI can estimate K+ ion levels from subtle changes in the ECG. Cardio-Net allows continuous, non-invasive, real-time monitoring of potassium, which can prevent death [68]

### 4.4. Sleep Disorders and Detection of ECG Pathology in Children

Preliminary evidence suggests that deep neural networks can estimate sleep phases from single-lead ECG data, potentially complementing more complex tests like polysomnography, though not replacing them [69]. The Apple Watch and AI could also be potentially useful as a medical tool for heart rhythm analysis in children. In 2023, the first algorithm based on AI and single-channel electrocardiograms was developed. The model, called Xception_v8, was tested on 48 children and showed high specificity (96.7%) with relatively low sensitivity (66.7%) in detecting a pathological recording. Further development requires more pediatric data and a comparison between the study group and the control group [70].

### 4.5. Advanced ECG Signal Reconstruction and Asynchronous Measurements

In addition, AI models enable efficient analysis of asynchronous ECG data from the watch. It is therefore possible to measure 3-4 ECG leads by changing the position of the watch and moving it to different parts of the body [71]. The Apple Watch Series 4 smartwatch, for example, has two electrodes—one embedded in the watch case and the other being a digital crown, in place of a button that is touched with a finger. The user takes three measurements by changing the position of the watch and finger. In this way, leads I, II, and III of a classic ECG can be reproduced, and the quality and accuracy of the measurements are comparable to a standard ECG [72]. Moreover, the addition of another six cardiac leads further enhances the reliability of ECG analysis and detection of acute coronary syndrome (ACS) [73]. This notably improves the chances of detecting an MI and opens the door to more advanced diagnostic smartwatch applications that are not limited to a single lead [71].

Although AI models can detect heart disease from reduced input data, accurate reconstruction of a full 12-lead ECG from only two leads still remains a challenge. In a study by Presacan et al., the AI-generated recording was found to have a significantly depleted signal, which proved to be of little clinical utility [74]. Interestingly, another model called ECGT2T—despite using a similarly limited signal from a smartwatch—was able to generate an artificial 10-lead ECG recording and detect Heart Failure with reduced Ejection Fraction (HFrEF) with a sensitivity close to 90%. This demonstrates that the effectiveness of AI does not depend solely on the precise reconstruction of the classic ECG, but on the ability of neural networks to extract hidden diagnostic patterns, even without having to map the entire signal [75]. Proof-of-concept studies indicate a potential for early hypertension detection using AI and a single ECG lead, though this approach remains experimental and requires clinical validation. However, studies with data directly from the watches are needed to put this method into practice [76].

## 5. Large Language Models (LLMs) in Cardiology

In recent years (2024–2025), there has been a rapid development of applications of large language models (LLMs) such as GPT-4V or the specialized ECG-Chat in cardiovascular medicine. An increasing number of studies confirm their potential as tools to assist physicians in daily practice, both in diagnosis and documentation. Multimodal Large Language Models (MLLMs), such as ECG-Chat, combine ECG signal data with natural language generation capabilities to automatically produce structured descriptions of ECG studies [77]. Such tools can provide valuable support in situations of staff shortage or high workload in emergency departments.

In studies evaluating ChatGPT-4V, the model was shown to be able to visually analyze ECG waveforms and provide answers to diagnostic questions, supporting clinicians in clinical decision-making [78,79]. Importantly, the system demonstrates not only the ability to read curves, but also to understand the clinical context—e.g., in relation to a patient’s medical history or medication.

In addition, systematic reviews indicate that LLMs have a wide range of applications in cardiology—from ECG analysis to medical record summarization to automatic generation of hospital discharges—although their effectiveness may vary depending on the clinical context [80]. These advances suggest that LLMs have the potential to significantly improve clinical workflow: automating report generation, supporting test interpretation, and providing conversational interfaces for clinical decision support tools at the bedside. As these technologies are further developed, their integration into everyday medical practice can bring real benefits in terms of efficiency AND safety of care and patient care.

## 6. Future Directions and Innovative Concepts

It is worth noting that some AI models for ECG analysis, such as the Mayo Clinic’s CNN-based model for detecting low ejection fraction, have already received FDA approval, confirming their readiness for implementation in everyday clinical practice [81,82].

**Figure 2:**
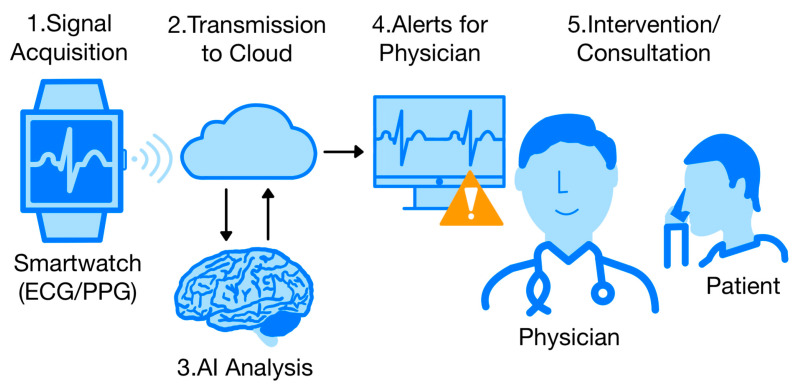
Data flow in modern AI-based cardiac care from ECG/PPG signal collection via smartwatch, through cloud-based analysis using artificial intelligence models, to clinical decision and patient intervention [own elaboration].

The integration of genetic data with ECG signals and other biometric parameters is gaining increasing attention [83,84]. Genetic variants (e.g., Single Nucleotide Polymorphisms (SNPs) associated with AF, HCM, or long QT syndrome) may provide an additional diagnostic dimension, and joint analysis with ECG features may facilitate the development of personalized risk stratification models [85,86,87,88,89,90,91]. In the long term, this could enable early detection of susceptibility to heart disease in asymptomatic individuals before clinical manifestations appear [83,84]. This approach is in line with the direction of precision medicine and may become the new standard for cardiovascular risk stratification. Figure 2 shows a diagram of modern cardiac care using AI and an ECG-assisted smartwatch.

A new and rapidly developing area is AI-assisted neurocardiology, which involves the simultaneous analysis of ECG and EEG signals [40]. Early studies show a correlation between heart rhythm and brain activity. The use of AI models to interpret these signals in parallel may open up new diagnostic possibilities, including in arrhythmia prediction or epilepsy diagnosis [92,93].

Modern AI models are increasingly integrating electrical heart signals (ECG) with imaging data, such as echo, MRI, or CT, significantly increasing their clinical utility. An example is LVH-Fusion, an algorithm combining ECG AND echocardiogram—showing that multimodal learning can better detect left ventricular hypertrophy than single-modality analysis [94]. Another study, DEEP RISK uses 12-lead ECG together with LGE-MRI images AND clinical data to predict ventricular arrhythmias, achieving an area under the receiver operating characteristic curve (AUROC) = 0.84—outperforming models based on a single modality alone [95]. A recent literature review highlights the fast-growing potential of multimodal AI in cardiology, although many models remain at the preclinical research stage [96]. Such systems stand a better chance of clinical implementation by combining functional and structural cardiac data—e.g., better detection of VHD, cardiomyopathy, or risk of sudden cardiac death. We suggest that future work should focus on validating these models in prospective trials AND assessing their impact on clinical decision-making.

**Figure 3:**
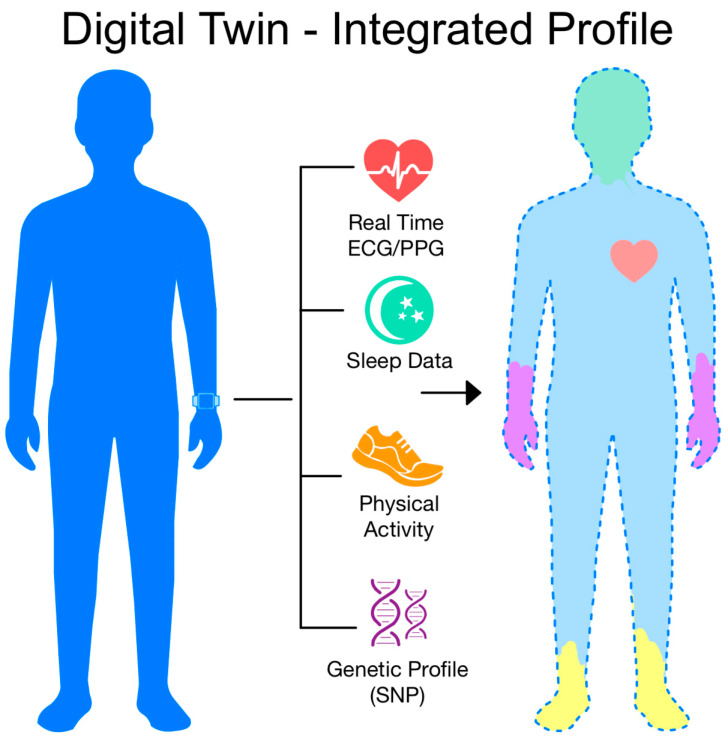
The concept of a patient’s ‘digital twin’—a dynamic, integrated health profile combining ECG/PPG data, sleep, physical activity, and genetics, synchronized with a real patient and analyzed by AI systems [own elaboration].

Among the most promising innovations is the concept of the patient’s digital twin. Such a model, built from given data such as ECG, PPG, HRV, sleep, and physical activity, clinical information, can be continuously updated thanks to AI. This creates the possibility of creating a dynamic patient profile that predicts future clinical events before they are visible to the clinician [97,98,99,100]. This could enhance approaches to monitoring, treatment, and personalization of care, although the scalability and clinical impact of such solutions remain to be demonstrated. As the boundaries between biotechnology, AI, and healthcare blur, there is a real opportunity to create a diagnostic ecosystem that not only detects disease but also supports its early identification and prevention. Figure 3 shows the concept of a patient’s digital twin.

One future direction for the development of AI models based on ECG data is the use of synthetic data, i.e., generated, for example, using Generative Adversarial Networks (GANs). This makes it possible to create realistic ECG or PPG signals even for rare diseases where the availability of real data is limited [101,102,103]. Furthermore, the use of a transfer learning approach—i.e., pre-training the model on large datasets and then tuning it for a specific population or even an individual patient—can significantly increase the efficiency and accuracy of the algorithm [104,105]. Such techniques not only overcome data limitations but also support the development of more personalized and error-proof digital diagnostics.

Recent advances also explore the use of wearable photonic devices, such as smart wristbands utilizing light-based sensors for the simultaneous assessment of cardiorespiratory parameters and biometric identification. These emerging technologies go beyond standard ECG or PPG sensors by leveraging optical components like photonic chips and near-infrared spectroscopy to capture subtle physiological signals [106]. In addition to monitoring, they can perform real-time biometric authentication, which may prove useful in ensuring data security and patient identification. For example, the wristband proposed by Li et al. employs an all-polymer photonic sensing unit, enabling continuous monitoring of respiratory rate, heart rate, and blood pressure, and achieving a biometric identification accuracy of 98.6% [107]. These novel systems may complement traditional ECG- or PPG-based methods and represent a new direction in non-invasive, continuous, and personalized cardiovascular diagnostics.

## 7. Integration with the Healthcare System

A critical aspect of implementing AI algorithms into clinical practice is their integration with existing medical record systems, or EHRs (Electronic Health Records). Data from wearable devices can be transmitted automatically and securely to the system used by the GP [108]. Such integration enables faster diagnosis, reduces clinical response time, and closes the gap between consumer and medical data [109,110]. However, appropriate interoperability standards (e.g., Fast Healthcare Interoperability Resources (FHIR), Health Level Seven International (HL7)) and regulatory compliance are needed here [111,112,113]. In order for AI-ECG to be used effectively in daily clinical work, it needs to be fully integrated with hospital systems (EHRs). For this, so-called open data exchange standards are needed, and the most widespread is FHIR (Fast Healthcare Interoperability Resources). FHIR-compliant AI systems can easily interface with the various software used in hospitals [114]. AI frameworks based on FHIR provide better interoperability between systems AND enable integration of AI-ECG with HER AND external applications. For example, FHIR-AI platforms have achieved a significant increase in interoperability (from 11% to 66%) in multi-centre deployments [115]. AI models can also act as a GP assistant, automatically analyzing background data and generating only relevant clinical alerts. The system could automatically refer the patient to a doctor’s consultation or directly to a specialist diagnosis, according to a clinical algorithm developed [116,117]. This type of solution would relieve the burden on PCPs and reduce the time from symptom to diagnosis, which could significantly improve treatment outcomes.

One of the main problems with the practical implementation of AI-ECG systems in hospitals is the so-called alert fatigue among medical staff. Studies show that more than 90% of generated alerts in decision support systems are ignored or turned off by doctors [115,118]. This can lead to genuinely relevant information being overlooked. Hybrid systems—alerts initially directed to the pharmacist or nurse, then escalated to the doctor, reducing the number of direct alerts to the doctor—can be a solution. AI-ECG should not only analyze the ECG signal but also realistically support the doctor in clinical decision-making. This is why it is so important that it works within CDSS systems, i.e., computer-based tools that provide actionable recommendations at the point of care. AI-ECG can generate point-of-care alerts, i.e., warnings that appear exactly where the doctor is making a decision—for example, at the time of prescription. This approach can improve efficiency [119].

In the context of public health, smartwatches, thanks to their ability to continuously monitor parameters, could act as primary prevention tools, identifying people with asymptomatic AF or prolonged QT before complications develop. An example is the Apple Heart Study, involving more than 400,000 participants, which aims to prove that mass detection of arrhythmias is possible and can lead to earlier medical intervention [120].

## 8. Challenges and Current Limitations

### 8.1. Regulation and Data Safety

The FDA (US Food and Drug Administration) does not classify smartwatches as medical devices, meaning that they are not subject to the same stringent requirements as traditional medical devices (e.g., ECG monitoring devices). Instead, smartwatches are treated as wellness tools that are approved more quickly under the Digital Health Software Precertification (Pre-Cert) program, which involves faster approval of technologies, but does not require full certification as for medical devices [121].

In the European Union, the Medical Device Regulation (MDR, Regulation (EU) 2017/745) classifies software intended for diagnostic purposes—including AI-driven ECG analysis—as a medical device. As such, these systems must undergo conformity assessment procedures, CE marking, and post-market surveillance [122]. In addition, data collected by smartwatches falls under the scope of the General Data Protection Regulation (GDPR), which emphasizes lawful processing, data minimization, purpose limitation, and the right to explanation in the case of automated decision-making [123].

Beyond regulatory requirements stemming from the MDR and GDPR, AI-based systems and wearables raise significant ethical concerns related to data privacy. Sensitive physiological data—such as heart rate, sleep stages, or respiratory parameters—are passively collected by smartwatches and fitness bands, often stored in commercial clouds, and can be vulnerable to cyberattacks, unauthorized access, or secondary use by third parties without the user’s informed consent [124]. Moreover, users typically lack a clear understanding of data retention periods, whether their data is used for further AI training, or if it is shared with external entities. Under European law, such data qualify as “data concerning health” under GDPR Article 4(15), requiring data minimization, transparent management, explicit consent, and rights to data portability and erasure after the original purpose is fulfilled [125,126]. These issues necessitate clear data governance policies, explicitly informed user consent, and system design aligned with Privacy by Design and Privacy by Default principles. Implementing anonymization, pseudonymization, encryption, and routine security audits is crucial in the everyday use of wearable health technologies [127].

Data security risks are also a concern. The mobile automated cardiac care ecosystem is based on a wireless sensor network involving personal devices (smartwatch, smartphone), cloud-based platforms, and external analytics systems. Each of these components must be adequately secured. Protecting data privacy and security is also about patient trust in modern digital medicine. This requires not only appropriate safeguards, but also transparent collaboration between technology developers, doctors, and users [3,5].

### 8.2. Technological Limitations

One of the challenges in using AI in wearable devices, such as smartwatches for ECG analysis, is the limited computing power and battery capacity of these devices. Traditional AI models are often resource-intensive, making it difficult for them to operate effectively in real time without having to send data to external servers. In response to this problem, tools have emerged, such as tinyHLS, an open-source package that allows AI models written in Python to be converted to energy-efficient hardware accelerators that can run directly on the device. Solutions of this type have the potential to break down current technical barriers and enable wider use of AI for arrhythmia monitoring in wearable devices [128]. A similar goal is pursued by the tinyML tool, which focuses on running optimized ML models directly on ultra-low-power microprocessors, such as those in smartwatches and fitness trackers. TinyML enables real-time detection of arrhythmias and other ECG abnormalities directly on-device, without the need for the cloud [129].

### 8.3. Algorithmic and Quality Problems

To fully exploit the potential of AI in smartwatch data analysis, the availability of large, high-quality, and diverse data is crucial. All AI models require it for training and validation. Projects such as TARGET that collect comprehensive, multidimensional health data are particularly valuable for the development of personalized, accurate AI algorithms [130].

It is also important to note that currently popular smartwatches such as the Apple Watch, Fitbit, and Samsung Galaxy Watch use PPG and a traditional heuristic (SP) algorithm to detect AF. However, this optical technology presents several limitations, such as intermittent monitoring (e.g., Apple only analyzes for 1 min every 2 h (i.e., less than 1% of the day)) and a high rejection rate (through movement artefacts or bad watch wear—up to 30% of signals). In addition, the study is heavily selective—data from people who are already ‘positive’ are often analyzed, so it is not known how many AF cases have been missed. Compared to this model, the DNN can ignore movement artefacts and learn AF-specific patterns. The DNN outperforms classical algorithms and allows almost continuous, non-invasive monitoring of heart rhythm [58].

The Siamese network is an example of an AI model, based on deep learning and part of the broader ML category. Although it is very good at detecting AF, it may not recognize other arrhythmias, does not take into account contextual data such as body position and movement and stress, and does not analyze very short episodes of AF that may be clinically relevant [54].

Instead of the classical windowing of data, it is better to recognize Human Activity (HAR) from smartwatch data for each signal sample separately (CEF), replacing the traditional fixed time window (SW) approach by using convolutional networks with different filter sizes—Multi-scale Convolutional Neural Network (MCNN) and intelligent feature weighting (attention) for channels and time space, which significantly improves performance, especially in data such as ECG or motion sensor data [131].

### 8.4. Bias and Generalizability

ECG data used to train AI models often come from demographically homogeneous populations—e.g., predominantly Caucasian males, which can lead to reduced diagnostic performance in women and ethnic minority individuals. Studies confirm that anatomical and rhythmic differences (e.g., ECG signal shape) can affect the performance of AI algorithms if this diversity is not taken into account. For example, Noseworthy et al. analyzed a CNN model to detect low ejection fraction (EF ≤ 35%) in a sample of almost 100,000 patients of different races and found that the area under the curve (AUC) remained high in all groups: non-Hispanic whites, Asians, black/African Americans, Hispanic/Latino, and American Indian/Native Alaskan [132]. However, another study evaluating a model predicting heart failure (HF) showed that accuracy significantly decreases in young black women—AUC lower than other groups, suggesting uneven model performance in a demographically dependent manner [133]. Therefore, it is crucial to report the effectiveness of models disaggregated by sex, race, and age, to test on separate, demographically diverse sets, and to implement debiasing techniques such as fairness-aware learning or adjusting decision thresholds for particular groups [134,135]. Such measures increase algorithmic fairness and minimize the risk of exacerbating existing inequalities in cardiac care.

### 8.5. Lack of Hard Clinical Evidence

The ECG is one of many elements in diagnosis. For a clinician to trust an AI decision, they need to understand how that decision was made in order to combine it with an overall assessment of the patient. AI algorithms—particularly deep learning models—often function as a ‘black box’—the clinician knows the outcome, but it is impossible to know the reasoning behind it [136,137]. Interpretable models, such as decision trees or machine learning, turn out to be easier to understand. The choice between specific AI models should depend on the problem, the available data, and whether the subsequent interpretability of the outcome is important [138]. For more complex models, such as neural networks analyzing ECGs, more sophisticated Explainable AI (XAI) techniques are emerging to allow the clinician to understand what influenced the model’s decision [4]. XAI techniques provide insights into the inner workings of the algorithms, which is crucial for clinical confidence and patient safety. XAI plays a central role in clinical acceptance, as clinicians require not only accurate predictions but also an understanding of why a model made a certain decision. XAI techniques help make deep learning models more transparent and trustworthy. An example is the so-called saliency maps, which visualize which parts of the ECG signal were significant in detecting pathology [139]. Another commonly used method is Gradient-weighted Class Activation Mapping (Grad-CAM), which allows visualization of which part of the ECG signal had the greatest influence on the model’s decision. In the context of medical images, heatmaps are used, while in the case of ECGs, the specific P-, QRS-, or T-wave segments that determine the classification are indicated. This is particularly useful for arrhythmia detection, as it allows assessment of whether the model focuses on clinically relevant features such as RR intervals or P- and T-wave morphology [140,141]. Another example is SHAP values, which indicate the influence of individual features (e.g., RR intervals, refractive amplitude) on the prediction outcome [142]. SHapley Additive exPlanations (SHAP) is a game theory-based technique that assigns a value to individual features for their contribution to model prediction. This makes it possible to determine which variables had the greatest diagnostic significance [143,144]. This approach increases the transparency of the model, without sacrificing its predictive power, and may play a key role in increasing physicians’ enthusiasm for AI algorithms. Despite generally promising results in the use of various digital health applications to detect AF, strong evidence that their use translates into better hard clinical outcomes, such as reduced complications or deaths, is still lacking. Therefore, further research in this direction is needed to confirm their clinical benefits and facilitate the introduction of these tools into daily practice [145,146].

At the same time, as the 2025 EHRA position paper shows, many AI studies in electrophysiology fail to meet basic reporting standards, such as data description, how to train the modellers, or how to record clinical trials. Of the 55 papers analyzed, none fulfilled more than 55% of the requirements from the 29-point checklist developed [26]. This means that, in addition to further clinical trials, improvements in the methodological quality and transparency of the analyses already carried out are also needed.

### 8.6. Differences in Adoption of Wearable Devices

Despite the rapid expansion of wearable health technologies, there remain significant disparities in their adoption across demographic groups. Studies show that smartwatch use is more prevalent among younger individuals with higher income and educational attainment. Conversely, older adults, those with lower socioeconomic status, racial minorities, and residents of rural or digitally underserved areas are significantly less likely to engage with wearable devices [147,148,149,150].

Such disparities pose a challenge to the equitable implementation of AI-driven cardiovascular diagnostics, potentially exacerbating pre-existing health inequalities. Additionally, limited digital literacy, cost barriers, or mistrust of technology may hinder user engagement and reduce the real-world effectiveness of these systems [151].

To ensure fair access and maximize public health benefits, future strategies should prioritize affordability, accessibility, and targeted education campaigns, especially among populations at risk of digital exclusion. Otherwise, the introduction of wearable-based diagnostics may inadvertently reinforce disparities rather than alleviate them [152].

## 9. Conclusions

Developments in mobile technology and artificial intelligence have significantly transformed approaches to cardiac care, enabling remote, non-invasive, and continuous monitoring of cardiac conditions. Smartwatches, supported by advanced AI algorithms, are showing increasing efficacy in arrhythmia detection, heart failure risk prediction, electrolyte disturbance assessment, and sleep quality analysis—with performance in some studies approaching that of traditional clinical methods, although variability in accuracy and validation strategies remains a concern. However, the technological limitations of the devices, the low specificity of the algorithms, and the need for standardization and integration into healthcare systems remain a challenge. Despite these barriers, developments—such as digital twin, federated learning, multi-modal analysis (e.g., ECG + EEG), the use of synthetic data, and personalized AI models—highlight the strong developmental prospects of this field. Introducing appropriate regulation, ensuring data security, and interdisciplinary collaboration are key to fully exploiting the potential of AI in everyday cardiology practice.

## Figures and Tables

**Table 1:** Summary of selected machine learning and deep learning (ML/DL) methods and their application in cardiac diagnostics. The legend of the abbreviations is given below: ML (Machine Learning), DL (Deep Learning), AF (Atrial Fibrillation), LAE (Left Atrial Enlargement), NSTE-ACS (Non-ST Elevation Acute Coronary Syndrome), MACE (Major Adverse Cardiovascular Event), HCM (Hypertrophic Cardiomyopathy), VHD (Valvular Heart Disease), CHD (Coronary Heart Disease), HA (Hear Attack), CHF (Congestive Heart Failure), AR (Aortic Regurgitation) [own elaboration].

Category	Abbreviation	Full Name	Use in ECG Interpretation	Bibliography
ML	SVM	Support Vector Machine	Heart rhythm classification, AF detection (also based on 1-lead ECG), LAE detection with raw ECG data.	[8,9,10]
	SVC	Support Vector Classification	Predicting MACEs and NSTE-ACS	[11]
	LASSO	Least Absolute Shrinkage and Selection Operator	ML technique for supervised learning. Used as a tool to select the best variables, among others, in HCM detection.	[12]
	MLP	Multilayer Perceptron	Detection of LAE when biological features are added.	[10]
	Extra Trees	Extremely Randomized Trees	Predicting the location of the source of ventricular arrhythmia.	[13]
	XGBoost	Extreme Gradient Boosting	Localization of the arrhythmia source (also based on 1-lead ECG).	[14]
DL	CNN	Convolutional Neural Network	Detection of VHD, detection of CHD, HA, CHF, angina, cerebral stroke on the basis of array data (without chronology information). Automatic classification of arrhythmias (also on incomplete ECG data). AR detection. Acts as a “black box”	[15,16,17,18,19]
	BNN	Bayesian Neural Network	Classification of arrhythmias (also on incomplete ECG data). Allows interpretation of the effect of each ECG feature on the outcome, shows decision uncertainty, which distinguishes it from CNNs.	[20]
	RNN:LSTM	Recurrent Neural Network: Long Short-Term Memory	ECG signal compression and reconstruction based on a limited number of leads or signal with noise.	[21,22]

## Data Availability

Not applicable.
